# Entangled in uncertainty: The experience of living with dementia from the perspective of family caregivers

**DOI:** 10.1371/journal.pone.0198034

**Published:** 2018-06-13

**Authors:** Els van Wijngaarden, Hugo van der Wedden, Zerline Henning, Rikke Komen, Anne-Mei The

**Affiliations:** 1 Research Group Care Ethics, University of Humanistic Studies, Utrecht, The Netherlands; 2 Research Organisation Tao of Care, Amsterdam, The Netherlands; 3 Faculty of Social and Behavioural Sciences, University of Amsterdam, Amsterdam, The Netherlands; University Antwerp, BELGIUM

## Abstract

**Introduction:**

Too often dementia care is still fragmented and unresponsive to the needs of people living with dementia and their family caregivers. To develop effective health care services, in-depth insight into the experiences of family caregivers is a prerequisite.

**Methods:**

This Dutch study is a qualitative interview study. The aim was twofold: 1) to develop an in-depth understanding of what it means to live with dementia and 2) to gain insight into what constitutes the art of living with dementia, both from the perspective of family caregivers. Data were gathered through 47 interviews with individuals and 6 focus group interviews. The analysis followed a phenomenologically inspired thematic approach.

**Results:**

The findings show that living with dementia can be understood as becoming entangled in uncertainty and isolation. The following themes illustrate this experience in various phases of the disease: a) Before the diagnosis: a growing uneasy feeling that something is amiss; b) The diagnostic disclosure: an uncertain and upsetting relief; c) Dementia at home: entangled in an isolated and exhausting life; d) Capitulation to relocation: torn between relief and grief. In addition, the study shows that the art of living with dementia is associated with: a) The ability to face tragedy; b) The discovery of meaning and dignity in the context of illness; c) Retaining a sense of connection and bond; d) The primacy of attention and recognition by others.

**Discussion and conclusion:**

Our findings show that dealing with what Boss (2011) called ‘ambiguous loss-experiences’ is one of the most demanding aspects of living with dementia. Based on the findings, we have developed a model that depicts how people handle contingency and seek balance along the continuum of facing and resisting. Our study shows that resilience in the context of living with dementia should not be understood as merely an individual mental ability, nor as a set of behaviours, but rather as a social-ecological enterprise.

## Introduction

The case in Box 1 (based on a true story) clearly illustrates the fact that–despite all efforts–there can be a huge discrepancy between the care demands of people living with dementia and available professional knowledge and support. 'Real dementia care’–as Herman puts it–does not seem to adequately take into account the depths of despair he and his wife are experiencing.[1]

**Box 1: *Case ‘Mismatch between professional care and real-life needs’***

Being part of a leading Dutch think tank we, together with several experts and one family caregiver, were commissioned to prepare a future agenda for dementia based on our national dementia strategy. During our meetings, the experts tried to convince each other of the importance of their specific expertise. Nothing special, that’s how things go in think tanks…

After a while, the family caregiver–named Herman–had not made any contribution to our discussion yet. Two years ago, his wife was diagnosed with Alzheimer's. Looking at Herman’s face, you could see discomfort, distress, and–not least–fatigue. I gently asked him to join our discussion and invited him to share his needs–or even better–*their* needs: “What do you think about the suggested ideas so far? What would support you?”
"You know,” Herman said, “I’m fine. Real care, as you describe it, no, we don’t need that."
“But how are you managing? How are you dealing with daily life?" I asked him
"Ah, is that what you mean…" Then Herman remained quiet, searching for words, unable to express himself.
After some encouragement, he started to tell us how life had changed enormously: All day, his wife clings to him in desperation, being nothing without him. Often, he feels trapped in the situation, completely deprived of his freedom, and utterly desperate, with no idea what to do. "But the real dementia care as you discuss today…"–Herman hesitated a bit and looked shyly around the table–"no, at the moment, we don’t really need that yet." [1]

The World Alzheimer Report (2016) shows that Herman’s case is no exception. On the contrary, the report states that “even when dementia is diagnosed, the care provided is too often fragmented, uncoordinated, and unresponsive to the needs of people living with dementia, their caregivers and families” [2]. An urgent question is therefore how to bridge the gap between professional views, theoretical models and therapeutic interventions on the one hand and real-life needs and care demands on the other.

This question is crucially important, as family caregiving for people with dementia is known to often lead to high levels of stress, depression and decreased health [3–9]. A recent Dutch study has shown that a large proportion of family caregivers suffer from complaints such as depression and fear [10]. Moreover, almost 40 percent of the family caregivers develop severe depressive symptoms within two years, some of them even having suicidal thoughts, resulting in a lower sense of competence and control [10]. Also, an Australian study showed that homicidal ideation is present among family carers of people with dementia [11].

Over the years, the difficulties and risks inherent to the role of family caregiver of a person with dementia [3–11] have been examined in numerous studies, emphasizing physical, social and behavioural functioning (e.g. coping and adaption strategies) which is undoubtedly important. To date, however, there have been few qualitative investigations into the experience of family caregivers who live with a person affected by dementia from an existential lifeworld perspective [12]. A recent study investigated family caregivers’ efforts to preserve the personhood of individuals with advanced dementia when they were moved to a long-term care facility [13]. Other previously published studies focused on the lifeworld of the spouses of persons affected by early-onset Alzheimer’s disease [14, 15], the grief experiences of children who have a parent with young-onset dementia [16], or the experiences of dementia for couples [17].

Thus, whilst some research has been carried out on existential aspects of the experience, to date there is a paucity of knowledge about the ongoing existential search for balance and meaning in the highly uncertain trajectory of dementia. To develop effective health care services for people with dementia and their significant others, we think it is a prerequisite to continuously listen closely to the voices of the people concerned in order to gain in-depth insight into their subjective experiences and focus on the question what it *means* to live with dementia. To that end in 2015 we launched the *Dementieverhalenbank*, a Dutch digital collection of narratives of people with dementia, their family and their caregivers. It is part of a bigger project—*Proeftuin Sociale Benadering Dementie—*which is an experimental learning and research laboratory for structural development, implementation and evaluation of new ideas, interventions, methods and institutional arrangements in dementia care (File. Background information project). Drawing on the insights of the *Dementieverhalenbank*, this current study attempts to deepen our understanding of the daily struggle of family caregivers. The aim was twofold: first, to develop an in-depth insight of what it means to live with dementia from the perspective of family members who were–to a greater or lesser extent–involved in the care of their loved one. Second, to gain insight into what constitutes the art of living with dementia. Starting from an existential perspective, important questions were:

✓ What does it mean to live with and care for a person with dementia?
✓ What are the essential needs and concerns of family caregivers?
✓ How (and to what extent) do they handle contingency and deal with the distinctive nature of grief and sadness experienced by the family members of persons with dementia?

## Methods

### Ethical approval

In 2014, the AISSR (Amsterdam Institute for Social Science Research) Ethical Advisory Board and the Faculty Ethics Committee have granted approval for the study ‘Dementia at home’ to the last author (number: 2014-AUSSR-3805).

### Data collection

This study is a qualitative in-depth interview study analyzing narratives of Dutch family caregivers of people with dementia, following the principles of a phenomenologically inspired Thematic Analysis Approach [18–20]. Data were gathered through interviews and focus groups, both of which were conducted in the context of the *Dementieverhalenbank*-project (File. Background information project).

In this study as well as in our whole project, we focus on the long period of ‘living at home with dementia’ [1]. In recent years, this period ‘at home’ has become increasingly demanding due to the fact that Dutch health policy places much more emphasis on personal responsibility and participation. This has an immediate effect on those living with dementia and their close ones. Since admission to a nursing home should be postponed as long as possible, next of kin are supposed to take full responsibility by engaging in care at home for the person with dementia.

With regard to the interviews, people signed up voluntarily. They were recruited through research advertisements. After applying, an interviewer made an appointment at the participant’s home. Prior to the interview, participants received an information letter outlining aim, procedure, privacy, contact details. Participants were asked to give written informed consent. Semi-structured in-depth interviews were conducted in people's home environment, using an interview guide containing an overview of the major topics to be covered by the interview (File. Interview guide). The guide has a dynamic structure to stimulate a free-flowing, explorative conversation [21, 22]. Interviews were audio-taped and transcribed verbatim. Observational field notes were made.

For the current study, the first author purposefully selected 47 interviews from this database [19, 23]. Selection criteria were: 1) information richness of the cases in relation to our research question and 2) diversity regarding the person with dementia, age, gender and demographic backgrounds [23]. These criteria follow from the assumption that experiential data in qualitative (phenomenologically inspired) research should be characterized by information richness and variation of cases [19, 20, 24]. In this specific case, ‘information richness’ turned out to be most crucial. Some interviews had little depth and/or did not provide much experiential description of day-to-day life. As they did not add to our understanding we decided to exclude those interviews. Furthermore, we aimed for diversity rather than similarity in our data, which explains our heterogeneous sample. The more variations the data reveal, the more likely it is to see patterns of essential meanings [20, 24]. In this case, we chose to include all available male respondents (whether partners or sons) and all respondents with a migrant background. We had quite a few interviews with daughters that highlighted similar issues. So we excluded some of them as they did not further enlarge our understanding. For an overview of the included participants, see Table 1.

**Table 1:** Overview of INTERVIEW participants’ characteristics.

Gender of the family caregiver	Participants *(n = 47)*
Male	15
Female	32
**Relationship to the person with dementia**	
Spouse (male)	7
Spouse (female)	9
Son *Of whom with a migrant background*	8 *(3)*
Daughter *Of whom with a migrant background*	19 *(5)*
Daughter-in-law	2
Granddaughter *Of whom with a migrant background*	2 *(2)*
**Cultural background of the family caregiver**	
Dutch	37
Turkish	5
Moroccan	2
Surinamese-Hindu	1
Iranian	2
**Age of the family caregiver**	
20–39	8
40–49	6
50–59	14
60–69	9
70–79	6
80+	4
**Age of the person with dementia**	
40–49	1
50–59	-
60–69	8
70–79	18
80+	20
**Marital status of the person with dementia**	
Married	32
Widowed	11
Divorced	2
Single	2
**Type of dementia**	
Alzheimer's disease	27
Vascular dementia	3
Mixed dementia	3
Frontotemporal dementia	4
Unknown	10

With regard to the focus groups, we organized two groups that both met three times, which has led to a total of 6 group interviews [23]. In September 2015 (with a two-week interval), the focus group interviews took place in a Dutch *Odensehuis*, a homely meeting place for people with dementia and family caregivers. The first group consisted of 6 people (3 men / 3 women). The second group consisted of 4 participants (all women). The median amount of time for the focus groups was 1:52 hours. Both focus groups were led by a moderator (IR) who encouraged disclosure in an open and spontaneous format. Each of the three meetings focussed on a specific period in the disease namely; 1) the period leading up to diagnosis, 2) the long period at home, and 3) the final phase. The discussion was structured around a set of carefully predetermined but open statements to create a well-regulated yet free-flowing discussion [23]. In addition to the moderator, a senior researcher of the *Dementieverhalenbank* (AMT) and an observer/researcher (LV) joined the moderator. Together they created an accepting environment that put participants at ease, allowing them to thoughtfully answer questions in their own words and think along with the ideas and experiences of other participants. For an overview of the participants’ characteristics, see Table 2.

**Table 2:** Overview of FOCUS GROUP participants’ characteristics.

Gender of the family caregiver	Participants *(n = 10)*
Male	3
Female	7
**Relationship to the person with dementia**	
Spouse (male)	3
Spouse (female)	6
Daughter-in-law	1
**Nationality of the family caregiver**	
Dutch	10
**Age of the family caregiver**	
50–59	1
60–69	6
70–79	2
80+	1
**Age of the person with dementia**	
60–70	2
70–80	3
80+	5
**Marital status of the person with dementia**	
Married	9
Unknown	1
**Type of dementia**	
Alzheimer's disease	8
Vascular dementia	1
Dementia with Lewy bodies (DLB)	1

Both for the interviews and the focus groups, the main aim was to gain in-depth insight into the lifeworld of family caregivers of people with dementia by gathering a broad range of ideas and experiences from an insider perspective.

### Data analysis

For the purpose of this study, the interviews were analyzed following an inductive, thematic approach [18, 23] which means that identified themes are strongly experientially driven and grounded in the data, rather than theory driven. The analysis consisted of different phases; first, the researchers tried to familiarize themselves with the data by repeated and active reading of the whole data set. Interpretation was discussed within the *Tao of Care*-team. Next, an inductive, bottom-up search was undertaken for themes related to the research question. Text elements were coded. Then codes were compared, combined and summarized in main themes and subthemes using the software program Atlas.ti 1.5.3 for Mac. In the next phase, the themes were reviewed in search of a coherent and valid pattern: the themes should form an accurate representation of all meanings evident in the data set [20]. Writing was not something that took place at the end, but an integral part of analysis, as the writing process itself deepened our understanding, clarified meanings and highlighted layers and polarities in the data [18, 25]. Findings were mutually discussed by all authors and within our research group. Intersubjective reliability was sought throughout the analysis process.

## Results

Below, we first depict the experience of living with dementia from the perspective of family members, who were all–to a greater or lesser extent–involved in the care for the person with dementia. Secondly, we provide a detailed description of what constitutes the art of living with dementia. Below, we have provided a thematic overview (Box 2).

**Box 2: Thematic overview**

**Experiences of living with dementia from the perspective of family caregivers:** Entangled in uncertainty and isolation Before the diagnosis: a growing uneasy feeling that something is amiss

- Growing suspicions and doubts
- A growing sense of disharmony

The diagnostic disclosure: an uncertain and upsetting relief

- A paradox of give and take
- Feeling trapped in a prospectless future

Dementia at home: entangled in an isolated and exhausting life

- A hidden and uncomprehended world
- Loss of equality and reciprocity
- Imprisoned in your own home

Capitulation to relocation: torn between relief and grief

- The inability to keep your promise
- Letting go and maintaining grip

**The art of living with dementia from the perspective of family caregivers:** Facing rather than resisting

- The ability to face tragedy
- The discovery of meaning and dignity
- Retaining a sense of connection
- The primacy of attention and recognition by others

### Experiences of living with dementia

From the perspective of family caregivers, living with dementia is essentially understood as becoming entangled in uncertainty and isolation. Despite all public attention for dementia, daily life at home is still experienced as a hidden and uncomprehended world. Without exception, respondents talked about loneliness and exhaustion. They felt trapped in a prospectless and uncertain future, increasingly losing a sense of equality and reciprocity. The themes below further illustrate the meaning of the experience of living with dementia in the various phases of the disease.

### Before the diagnosis: A growing uneasy feeling that something is amiss

#### Growing suspicions and doubts

The family caregivers under research stated that in the early stages, they had regularly had an indefinite feeling that something was amiss: *“Somehow*, *I knew that something was off*, *but I couldn’t put my finger on it*.” Or: *“I had a gut feeling that something was wrong*.*”* Some vague clues strengthened increasingly uncomfortable feelings. Initially, they had tried to explain away unusual behaviour. Forgetfulness, disorientation or getting lost when running into unexpected road works; in a way, it can happen to anyone. But gradually respondents had to acknowledge that things could not be explained, and most began to suspect that something was seriously wrong. For example, one respondent found her husband urinating on a chair in the bedroom. Others became suspicious when confronted with a disorganized administration, missed payments, late charges, or even serious financial debts.

In some cases, it was merely the partner’s reaction that evoked suspicion. One respondent (i_55) had asked her husband to make some tea. When she suggested that he had forgotten the teabag, he became angry. He was absolutely sure that he had not forgotten it, he simply denied that there was no teabag. She was shocked; not because of his forgetfulness–she had noticed that before–but because of the way he dealt with this incident: the denial and the anger. Another respondent (i_12) had a similar experience. She was hiking with her husband in the Ardennes when they got lost. Previously, this would not have been a problem, but now, her husband panicked and began to cry in desperation: *“A sixty-year-old man*, *in a total panic…”* Again, it was not the fact that he did not know the way like he did before. Rather, it was the manner in which he reacted which made her think and gave her a profoundly uneasy feeling.

#### A growing sense of disharmony

Many respondents told us about the way their relationships gradually lost harmony. Situations escalated more often. Some felt that, at times, they had become like rivals, constantly contradicting each other. The normal course of things became increasingly threatened by all the upset at home. Many respondents muddled through for years—behind closed doors—without an explanation. Often, they felt torn between anxiety and self-doubt. In most cases the gradual nature of the process had blurred what was really going on. Not only the person with dementia had changed, but the significant others also (often unwittingly) altered their attitudes and displayed ineffective (and sometimes disruptive) behaviour. Gradually their tone had become curt, annoyed and unfriendly.

In retrospect, many respondents indicate that this confusing, indefinite situation was probably the most difficult and heaviest episode of living with dementia. Gradually, the disease had slipped into their lives, entangling them, powerless, in this new phase of life.

### The diagnostic disclosure: An uncertain and upsetting relief

#### A paradox of give and take

For most respondents, the disclosure of the diagnosis of dementia was a paradoxical experience; it gives and takes away. On the one hand, most family caregivers had experienced the diagnosis as closure. It gave them a sense of relief. Finally, a very uncertain and unexplainable episode had come to an end: *"The clarity it brings is really appreciated and valued*!*"* One respondent (i_54) said that she cried very hard when her husband’s diagnosis was disclosed. The doctor asked whether she was shocked. *"No*,*"* she replied, *"I'm just so relieved* …*"* Finally, she understood why her husband put nutmeg on his steak instead of pepper. Many respondents said that–after the disclosure–their attitude towards their loved one had changed profoundly. All the weird things and frustration could now be explained, resulting in increased understanding and strengthened resilience.

On the other hand, most respondents felt they were left in a state of uncertainty, having no idea about the course of the disease and what they could expect of the future. One man (i_6) said irritably:

The neurologist just announced: ‘It’s refractory so you don’t have to come back.’ And to me, he simply said: ‘From now on, you can completely focus on the caring.’ Well, I thought, that’s nice… not! We both cried bitterly.

From one moment to the next, respondents were given a new role–namely of family caregiver–without having any idea what this role entailed. Many respondents were particularly annoyed by the lack of information. Some presumed that the doctor had bitten his tongue in order not to upset them. One respondent (i_49) said:

I wished the doctor would have given us a calendar, or at least a description of the process, something to hold on to, something that might provide a little steadiness.

The disclosure of dementia was also accompanied by a deep existential uncertainty. It took away hope and trust in the future. Most respondents mainly associated dementia with a humiliating, progressive process, inadequate or poor care, dependence and social exclusion. To express their discomfort, respondents used words like: *“It felt like I was hit with a sledgehammer”* and *“a declaration of a plain*, *unvarnished truth about our future”*.

After the disclosure, a small minority or family members chose not to disclose the diagnosis to the person with dementia. Some feared the anger of their loved ones, others wanted to protect them against pain and disappointment. Rather than providing clarity, they assumed that the diagnosis would only increase stress and exacerbate the symptoms. One respondent (i_30) related that after the diagnostic examination, she asked the doctor whether the disclosure could take place by telephone. *"I don’t want my mother to hear it*,*"* she told the doctor. The doctor agreed and her mother was not informed. Another respondent (i_33) wanted to save her father from unnecessary distress and suffering. "*He has witnessed the deterioration of his uncle*, *and the trouble that came with it*. *I’m not going to say*: *you’re in the same boat*. *I just don’t want to do that*.*”*

In some cases, respondents regret sharing the diagnosis too quickly with their network. One woman (i_57) told: *“There was not much going on yet*, *and he was still saying very reasonable things*. *But immediately*, *he was no longer taken seriously by his friends and family*.*”* In the eyes of others, the diagnosis instantly took away his competence.

#### Feeling trapped in by a prospectless future

Most respondents perceived the future as hopeless, lacking any perspective for their loved one, but also for themselves. Some conceived the diagnosis as a death warrant: *“It’s a silent killer*, *there is no cure or stop*.*”* Others had experienced the disclosure as an announcement of a forced relocation to a nursing home. A way to safeguard their loved ones–and allay their concerns about the future–was to promise them that they would never allow them to end up in a care home. One respondent (i_12) stated: *"I remember*, *I reassured him*: *‘Absolutely*, *I will always take care of you*, *as long as I can*.*’”* In retrospect, though, she (as well as others) realized that she had no idea of the intensity of the promise they made.

### Dementia at home: Entangled in an isolated and exhausting life

#### A hidden and uncomprehended world

After the disclosure, most people intended to *“make the most of it”*. Respondents tried to remain optimistic and adjust and lower their expectations of life. The attitude of ‘making the most of it’ turned out to have a side-effect. Many respondents said that–while they courageously tried to continue life as they had known it–outsiders often got the impression that everything was fine, saying: *"Fortunately*, *it is not too bad yet''* or *“He is still looking good*!*”* While these remarks might be well-intentioned, caregivers often felt misunderstood, unrecognized, and deprived of the opportunity to share their struggles: *“They simply have no clue”*. Others were just mainly frustrated: *“They just don’t understand*. *Actually*, *I think they don’t want to understand* … *"*

Most people with dementia and their spouses became increasingly isolated. Their world was getting smaller and often they felt discarded. Not uncommonly, friends became reluctant to visit them. Sometimes because they felt embarrassed by the changed situation. Others were shocked, unable to deal with it, having no idea what to talk about, and how to find the right attitude. A few people even got angry. One respondent (i_8) explained how she got involved in a quarrel because of her partner’s behaviour: They were invited for a dinner at a friend’s house, but at the last moment, her partner simply refused to join her. Shortly after, something similar happened on a birthday. Three minutes after they arrived, her partner became very restless, immediately wanting to leave again: *“People just don’t understand it and take it personally…"*

Simultaneously, caregivers of people with dementia became reluctant to go on a day trip, afraid that they would become involved in awkward situations. *“What if you meet a friend and she doesn’t recognize him*?*”* Several respondents talked about embarrassing situations such as licking a plate in a restaurant, thumb sucking in public and sudden incontinence. A man (i_6) talked about an uncomfortable episode with his wife. They were relaxing in a beach pavilion. Because his wife was sitting in a draught and the owner did nothing to fix the source of the chill, she called him *“a dick”*. In his words:

Within two minutes, the guy showed up at our table—quite irritated—saying ‘if you have something against me, then say it out loud to me now!’ I offered him a beer, but he refused, remaining angry. But you see, I’m not going to explain…

Respondents’ stories show that caregivers are often hesitant to specify and explain the dementia in such situations, because they feel it is private or hurtful to their partner to disclose information about the disease.

#### Loss of equality and reciprocity

In many cases, people with dementia and their loved ones not only lost connection with friends, but also with each other. According to the respondents, one of the most difficult things is the gradual sense of detachment. Several respondents indicated that gradually they witnessed their loved ones drifting away from them. *“It’s a sort of ongoing grieving process*.*”* One respondent (i_22) said that previously, she and her partner watched movies, read books and newspapers, and afterwards they would have heart-to-heart talks. But lately, her partner lost the ability to follow even a basic storyline. *“Basically*, *we have nothing to share anymore*,*"* she sighs, *"more and more*, *you feel alone*.*"* Physically, the person with dementia is still there, but mentally and emotionally he or she is no longer present in the same way as before. This ambiguity is often experienced as very confusing.

A young woman (i_34) illustrated how painful this growing sense of distance can be. She had suffered from breast cancer. Due to the chemotherapy she lost her beautiful long hair, which made her feel really bad. After the chemo, her hair started to growing back slowly. Then her mother asked: *"Love*, *why did you have your hair cut*? *You had such beautiful hair*.*"* She reacted very angrily: *“Please mom*! *You do realize that I was seriously ill*, *don’t you*?*”* A little ashamed her mother answered: *“Oh yes*, *sure*, *I do know that* …*”* But later that day, she asked again why her daughter had had her hair cut. *“Then I decided to just leave it* …*”* However, the unintentionally hurtful comments strengthened a sense of distance and misunderstanding between her and her mother. Close, reciprocal contact belonged to the past now.

Over time, the person with dementia becomes increasingly dependent on his spouse or children, which deeply affects the relationship. Many respondents lamented their fate by saying: “*Sometimes I think*: *who is he*? *My husband or my child…*?*"* A daughter (i_18)—who struggled to combine the care for her mother with raising her two young children and a job—said: *“Sometimes I just don’t know how to handle things*. *It’s like I have another child to care for*.*”* Using a child metaphor, she describes the change in their relationship: a child needs guidance and is dependent on its mother. It also illustrates the resulting distance. One man (i_6) put it quite bluntly:

My wife isn’t my wife any longer. Basically, she has become a toddler with speech difficulties. We don’t make love anymore. I’m really losing the desire. Just because she’s not my wife anymore.

While the sense of distance and detachment was predominantly present in respondents’ stories, in some cases relationships changed for the better because of the dementia. One respondent (i_14) said that her relationship with her father had been somewhat strained. Since he had been living with dementia, she dared to put her arm around him and express her love to him. *"For me*, *it's very nice to spend time with my father now*,*"* she said. "*I cherish the intimacy that has developed between us*. *Perhaps that's why I think dementia is not too terrible*.*"*

#### Imprisoned in your own home

Behind closed doors, caregivers are increasingly preoccupied with caring responsibilities, which has a huge impact on their own lives. For most, as time goes by, there is no such thing as an ‘own life’ anymore. One respondent (i_33) recounted: *"At one point*, *I was so busy taking care of him that I had no activities other than him*. *He was my job and he was my hobby*.*"* Twenty-four hours a day, caregivers felt burdened with care and housekeeping. Most partners noted the total exhaustion they suffered as a result of this. Children who were not physically present all day also often felt permanently mentally involved and overloaded.

Besides, caregivers had to be constantly alert in order to protect their loved ones from incidents, checking, for example, that he did not use shaving cream instead of toothpaste. Most respondents felt absorbed by a project that lacked all perspective, using phrases like: *"I became a prisoner in my own home*.*"* One person (i_49) added: *“There’s an important difference though*. *You know*, *prisoners know when they will be granted release*. *But I have no idea*. *How many years to go*? *I find that very difficult*.*"* In particular, the oldest respondent said that, slowly, the hope for “a life after caregiving” faded away.

Some respondents said that the combination of hopelessness and solitude resulted in them secretly ideating about their partner's death. Partly to end their partner’s suffering, but also to end their own misery. One respondent (i_10) expressed herself in these words: *"I don’t have a husband anymore*. *I’m not allowed to say it out loud*, *but it would be better if he dies*. *It may sound sad*, *but a widow is better off than me*.*”* Another woman (i_48) put it like this:

I know it’s not right, but sometimes I’m so angry, I just cannot deal with it anymore. The ongoing demanding care. Constantly putting yourself on hold. There are moments that I’m overwhelmed with fatigue. When I drive home, I sometimes fantasize about his funeral.

Fantasizing about the death of the person with dementia is experienced as a taboo and a way of letting the person down, but also as a form of mental liberation. For some, it seemed to be a way to deal with a prospectless and threatening future, imagining the end of the experienced imprisonment.

### Capitulation to relocation: Torn between relief and guilt

#### The inability to keep your promise

After years of caring responsibilities, many respondents reached the point that they could no longer handle the situation. A severely disrupted day and night rhythm, clashes and conflicts, domestic accidents; they gradually felt an increasing strain completely overburdening them physically and emotionally. Due to earlier “naive” promises to never allow their loved one to end up in a care home, they tend to postpone the relocation to the last moment. Several respondents explicitly mentioned that it brought them deep relief that a professional had taken the decision that relocation to a nursing home had become inevitable.

Caregivers often felt guilty when they had to give up, calling themselves ‘egoists’ who put their own rest before the welfare of their partner or parent. Placement in the nursing home is considered ‘putting away’ or ‘abandoning’ their loved one. One respondent (i_33) said: *“His whole life*, *he has worked very hard to support us*. *And now*, *when there’s practically nothing he can do anymore*, *I feel we are discarding him*.*”* Another respondent (i_54) talked about her inability to keep her promise: *“All these years he asked*, *please don’t let me down*. *That's the theme of his life*, *feeling abandoned*. *And with my hand on my heart I promised him*: *‘Of course*, *I will never let you down*.*’ And look what I’m doing…”*. Some literally compared relocating to a divorce. The sense of distance and detachment in the relationship had now gained a spatial dimension as well.

#### Letting go and maintaining grip

After the relocation had taken place, most respondents experienced a sense of relief. However, the relocation to a nursing home did not necessarily mean that the feeling of overload decreased. Some professionals seemed to lack the expertise respondents had gained in dealing with the specific person with dementia, which regularly lead to friction. One lady (i_48) said that she found it rather complicated and time consuming to pass on her knowledge to the professionals in the nursing home. *“Those people don’t know him* … *they just have a new customer*. *But I have the user manual*, *you know*, *I know my husband quite well*. *I know how he responds*.*”* Besides, she criticized the communication: *“Sometimes*, *I had to tell the same story over twenty times*.*”* It took ten months before she felt that things started to go better.

### The art of living with dementia

The experience of uncertainty, ongoing loss, hopelessness, growing detachment and exhaustion associated with dementia was (to a greater or lesser extent) present in all stories. What varied was the way people were affected by these feelings. While some were dominated by anger, disappointment and resistance, others felt less severely threatened and somehow seemed to know ‘the art of living’ with dementia, at least to some extent. Yet others displayed ambivalence: they constantly shifted between resisting and facing their struggles. We will now provide a detailed description of what the ‘art of living’ with dementia for the family caregivers under research entailed.

#### The ability to face tragedy

Some caregivers strongly resisted dementia. However, there were also many respondents who were able to face the situation, at least to a certain extent. Not that they trivialized the tragedy and suffering, but they had somehow ‘chosen’ to give up resistance and tried to make life with dementia as bearable as possible for their loved ones and for themselves.

You constantly switch between 'how am I going to manage?' and 'just do it!'. And yes, the emphasis is on ‘just do it’. It’s best not think too much about why you do it, you just do it, and yes … that it’s going to wear you out, that it will completely exhaust you, that’s true! But it is what it is… He did not ask for it, neither did I… so you have to deal with it. And as long as there is no medication to solve the problem, you’ll have to go on, and preferably in the best way possible. (r55)

Others said that it was not so much a choice, but rather an attitude, a character trait. *“You know*, *I don’t have any perfect idea about how life should be*, *or what a good life consists of*. *That’s easier*.*”* (r22) Most just try to make the best of it by creating an enabling and supportive environment where their loved one feels valued and understood. One lady (r27) stated:

A sense of rest and peacefulness, I think that’s most important, and to accept him the way he is. That he is not a failure. My husband sometimes says, I have become an unreliable, dodgy guy. Then I just tell him: No, not at all, you are a very reliable person, but Alzheimer’s took up residence in you, and he’s the one that's unreliable, not you. (…) He just needs confidence. So, I let him do as much as possible. He still does the dishes. Well … they’re not always clean or put in the right place, but what does it matter? And, if something's really dirty, I'll do it again when he’s not there. What matters is that he feels he is still valued.

Others emphasize the importance to not take it all *‘so bloody seriously’*, and that it is better to laugh about the situation and see the tragicomic side of it. One lady (r25) said: “*I just want to remember the nice things*. *Of course*, *many times things happen that aren’t fun at all*.*”* Then she related a nasty toilet visit and the way they dealt with it: *“You know*, *he was completely covered in poop*. *And then*? *We just had a really good laugh about it*. *(…) Just about the whole situation*.*”* Several people said that they did not attend support meetings for fellow caregivers, because they did not like the focus on suffering, worries and concerns. One caregiver (r25) said: “*Whining*, *whining*, *and more whining*. *Too heavy-hearted*, *you know*. *I was wondering*, *what are they talking about*? *Am I so different*? *Perhaps their situations are more serious*? *I can’t judge that* …*”*

Respondents with a migrant background (11 in total) seemed to have distinctive views of acceptance. According to one respondent (r42), in his Hindu-Surinamese culture, dementia is viewed as their fate; what matters is whether you can face it and resign yourself.

Well, you cannot do anything about it. When it happens, it happens. It’s not in your hands. Of course, it's hard, you didn’t ask for it. But these things are not up to us. You may be angry, but that’s just not helpful. You must learn to live with what you are given.

Several interviewed migrants did not view dementia as a medical disease. Among the Turkish respondents, the characteristics of dementia are more commonly known as *bunamak*, by which they mean a quite normal and accepted state of forgetfulness that is associated with aging: *“We use the word bunamak for situations in which you say*, *that old one* … *he’s just worn out and old*. *It’s just part of aging*, *we accept it*.*” (r36)* Other migrants did call it a disease, but they also emphasized acceptance and the duty to care. “*We might not like it*, *but we are obliged to accept it*. *It happens*, *and they are our parents*. *(…) …whether they are crazy or well… we have to accept them*, *protect them*, *and keep them with us” (r58)*.

Caregiving is seen as a kind of selfless reciprocity, a way of mutual care. Formerly, their parents have provided them with care and opportunities such as education. Now, it is their turn. In some cases, children care for their parents for many years. Not only by providing the daily care, but also by filling in forms, accompanying them to medical appointments, and translating information. Some respondents even moved into their parents’ house to provide the daily care. In most cases, relocation to a nursing home was not seen as an option.

No, no. That’s not our culture. I would never want to do that to my mother. She has always taken care of us, and now it's our job to take care of our parents. I will never send her away. And if it gets worse? Yes, we will have to see what will happen and what to do, but really, to a nursing home or something like that, no, that is not negotiable. That will never happen…

#### The discovery of meaning and dignity

Most respondents found it very important that the person with dementia felt that they were still a valued part of the family and of social life. While several respondents emphasized the meaninglessness of the life of the person with dementia, many respondents recognized meaning and value in the situation. One respondent said (r3): *It’s mainly about your conception of humanity*. *The recognition of full personhood*. *That they are welcome and accepted*. *For me*, *it’s about looking for the beauty*, *the dignity*, *and also the truth (…) because in a sense*, *they don’t beat around the bush”*

Most respondents talked about feeling a kind of responsibility; they should “do” something to confirm dignity and meaning in the lives of their loved ones. Some indicated that it was mainly about “maintaining” someone’s dignity, or “searching” for dignity, or “assigning” meaning and a sense of dignity to the situation by taking a loving attitude. A son, who took care of this father (r47) stated: *“Of course*, *I saw the panic and the fear*, *and the poop on the wall*, *all those unworthy things*, *but you know*, *I could add some dignity* …*”* Another son said about his mother (r39):

That’s the reason why I want to tell people that it’s not all doom and gloom. I'm not going to deny that it's tough, because it's definitely hard, but in all this hassle, I felt very much strengthened by the fact that I could see what was happening; that her mask was removed and that she became so pure …

Some relationships became more intimate; relations between spouses, between parents and children, or between grandparents and grandchildren. For example, one daughter (r26) talked about a deepening of the relationship both with her mother and her father. During the process, she got to know them in a different, more intense way:

Yeah, the bright side of my mother’s Alzheimer’s was that she became increasingly sweet [laughter]. Really, she was so sweet and so thankful if we were just sitting cosily together, just holding hands was enough. That’s a very precious memory…. you know, before, my mother was not so cuddly.

…I really admire my father for his loving care. My father had, let’s say, two faces. He could be very hard … but also kind of sweet. The love, the care he showed for his wife was just amazing… She always looked so good. With his huge, rough working man’s hands, he put a little eye shadow about her eyes, clipped on a pair of earrings, just like she did in the past (r26).

Several respondents said that living with dementia has really been *“an enriching school”* for them. It taught them *“to deal with fears”*; *“to not run away from difficulties but rather face them”*; to enjoy *“the satisfaction of small pleasures”*; and to *“slow down the speed of living”* and learn to *“live in the moment and be less preoccupied with the past or the future”*. Some stated this attitude might require a certain view of life. One respondent said: *“You have to become at peace with the situation*, *and perceive it as a different opportunity to be happy and content*. *But*, *I guess*, *you probably have to have this attitude already*, *at least a bit*.*”*

#### Retaining a sense of connection

Above we described that one of the most painful and unsettling experiences for significant others is the growing sense of detachment and loss of reciprocity. Without trivializing this experience, it turned out that some respondents were still able to keep a sense of connectedness with their loved ones, even in more advanced stages of dementia: *“I’m trying to keep our togetherness to the very end”* (r28). Maintaining a sense of connection required an ongoing effort to be on the same wavelength as their loved ones. The ability to calmly attune was seen a prerequisite. One respondent (r11) said:

Above all, it means that you have to take it easy. Not ask several questions at once, like: ‘Dad, can you put on your shoes and grab your coat?’ He just cannot handle that. So, it’s first the shoes, and then the coat. When I'm patient, he does fine, but I had to learn.

Another man (r28) said that after the disclosure, he felt absolutely devastated. However, over time, he tried to move forward and continue the activities he and his wife had always engaged in. When he organized drinks with friends, his wife had a slightly different role during the event: *“She no longer mingles*, *she just enjoys drinks and nibbles* …*”* Respondents talked about the need to stop reprimanding the person with dementia. *“You shouldn’t correct him all the time*.*”* Others emphasized the need to resist thinking in terms of human degradation. One lady (r8) talked about her partner licking a sausage at the butcher’s. At first, she felt deeply embarrassed for her, but after a while she realized: *“Basically*, *if she doesn’t mind*, *why should I*?*”*

Because the development of the disease is unpredictable but progressive, staying connected to one another requires a constant, renewed effort to attune. Some compared it with a sort of *“adventure”* or an *“unpredictable game”*. They had to learn to live with sudden changes of mood and erratic behaviour and the impossibility of becoming familiar with the situation. Indeed, living with someone who is affected by dementia can be seen as a journey in which you constantly have to find a new balance and refamiliarize yourself with new ways of communication and adjust your expectations. Caregivers had to surrender themselves to the unknown.

#### The primacy of attention and recognition by others

For all participants, caring for a person with dementia emerged as a very demanding process. Without exception, they felt heavily burdened with care responsibilities, and longed for support and supportive information. Professional caregivers (i.e. case managers, home healthcare workers, or GPs) could play a significant role in supporting participants and enabling them to provide the necessary daily care. However, in practice, respondents told many stories about (unintended) mismatches.

In respondents’ views, case managers and physicians lack knowledge about daily life with dementia. They frequently felt more knowledgeable than the professional caregivers. One respondent (i_22) stated: *"I've had four case managers*, *but in all cases*, *I knew more about it than they did*. *Once one of them said very excitedly*: *Wow*, *it’s like you're my case manager*.*”* Others suggested that their case manager is more of a burden than a support, due to their limited task interpretation (i_18): *“When we ask if she can look in my mother’s fridge during her visits*, *she replies that that’s not part of her work”*. Besides, many respondents felt entrapped by regulations in care services, which were experienced as intimidating, bureaucratic and incomprehensible. To describe this, people use words like *“drama”*, *“hassle”*, *“fight”* and *“powerlessness”*.

Despite much criticism, there was also praise for the gained support. For example, respondents were thankful for having a case manager who knew their way around the health care system and legislation. In other cases, family caregivers highly appreciated a timely signalling of the need for support before they themselves realized or dared to express their needs and concerns. Essentially, respondents described a good professional caregiver as an understanding person who fully acknowledged the tragedy and the uncertainty and unpredictability of the disease. It was a professional who was prepared to take time and who was attentive to their personal experiences.

…our case manager, well, she’s worth her weight in gold. She accompanies me, and at some point, she said: ‘This is just too much, you cannot handle this anymore. We are going to arrange something.’ Basically, it’s just that I have someone to talk to. (r14)

Respondents’ stories showed that, for them, an attentive attitude of the professional caregiver is far more important than the actual intervention. When caregivers asked for more information, it primarily seemed to be a call for recognition of the deep uncertainty they had to deal with. Besides, it was also evident that support was considered to be good when provided in mutual consultation. Family caregivers wanted to collaborate with professional caregivers as partners who do justice to their experiential knowledge by taking it fully into account.

## Discussion

In this study, we first sought to describe what it means to live with dementia from the perspective of family members who were involved in the care of their loved ones. Our dataset of qualitative interviews and focus groups showed that living with dementia is to be understood essentially as becoming entangled in an uncertain and isolated life. Family caregivers felt trapped in a prospectless future, as they increasingly lose the sense of equality and reciprocity, and struggle to deal with subsequent experienced contingency and ambiguity without any guarantees except the certainty of death. Due to the unpredictable but progressive development of the disease, staying in connection with the diseased person requires an ongoing effort to attune. Family caregivers were confronted with accumulated losses. They constantly had to find a new balance, refamiliarize themselves with a changed situation. Witnessing this relentless progression of the disease and dealing with the accompanying uncertain changeability shows itself as a very demanding process for close family members, as well as for their loved ones affected by dementia.

Secondly, we aimed to explore how family caregivers handled contingency and dealt with dementia and the related ongoing loss experiences. Our study illustrates that the experiences of the family members can be characterized as a complicated grieving process in which they have to deal with so-called ‘compounded serial losses’ [26] which are numerous and cyclical in nature. Blandin and Pepin developed a theory of ‘dementia grief’ [26] to interpret the nature of loss and grief in dementia. Our study supports their theory, as well as the theory of ambiguous loss [27–29]. It illuminates the ambiguity of the accumulated loss experiences in the sense that the ongoing changes are unstable, fluctuating, and lacking all clarity [16, 27, 28, 30–32]. Our findings clearly show that dealing with this ambiguity is one of the most demanding aspects of living with dementia.

Interestingly, while the unsettling experience of ongoing loss was common in respondents’ stories, the ways it affected individual family caregivers were far from uniform. Some respondents found it almost unbearable, not only for the person with dementia but also for themselves. Other respondents, however, showed considerable resilience. To a greater or lesser extent they were able to give up resistance and face the difficult task of balancing the experiences of dementia as part of their life. Despite the demanding effort it required, they somehow committed themselves to the situation and were still able to recognize meaning and value in life. Our findings underline the importance of constantly seeking new ways to attune, connect and reconnect. As Taylor stated: the main concern should not be whether the other is still able to recognize you, but rather whether the person is still granted (social) recognition.[33].

If some seem to be better able to face and adapt to the contingencies of dementia than others, an interesting question is how this difference can be explained. In recent literature, this question is often considered from either a burden perspective [3–8, 10], or a quality-of-life perspective [9, 34–38], commonly starting from a psychological-behavioural paradigm. They focus on 1) predictors of burden such as stress, depressive and/or grief symptoms; 2) risk factors such as behavioural problems in the care recipient, conditions of the disease and/or poor economic situation; 3) coping strategies of the caregiver; or 4) quality-of-life domains such as health and vitality of the caregiver, supportive social environment, and other resources such as own projects. Our findings suggest that the explanation of how family caregivers deal with their demanding tasks and roles lies in a combination of all these aspects. However, our results go beyond this psycho-behavioural symptomatic approach by taking a more holistic lifeworld approach that considers both the tragic burden-experience, as well as the perceived quality of life, without focussing on one side or the other. We suggest that such a lifeworld understanding contributes to our in-depth understanding of the every day reality of family caregivers, and the huge impact it has on their lives.

**Fig 1:**
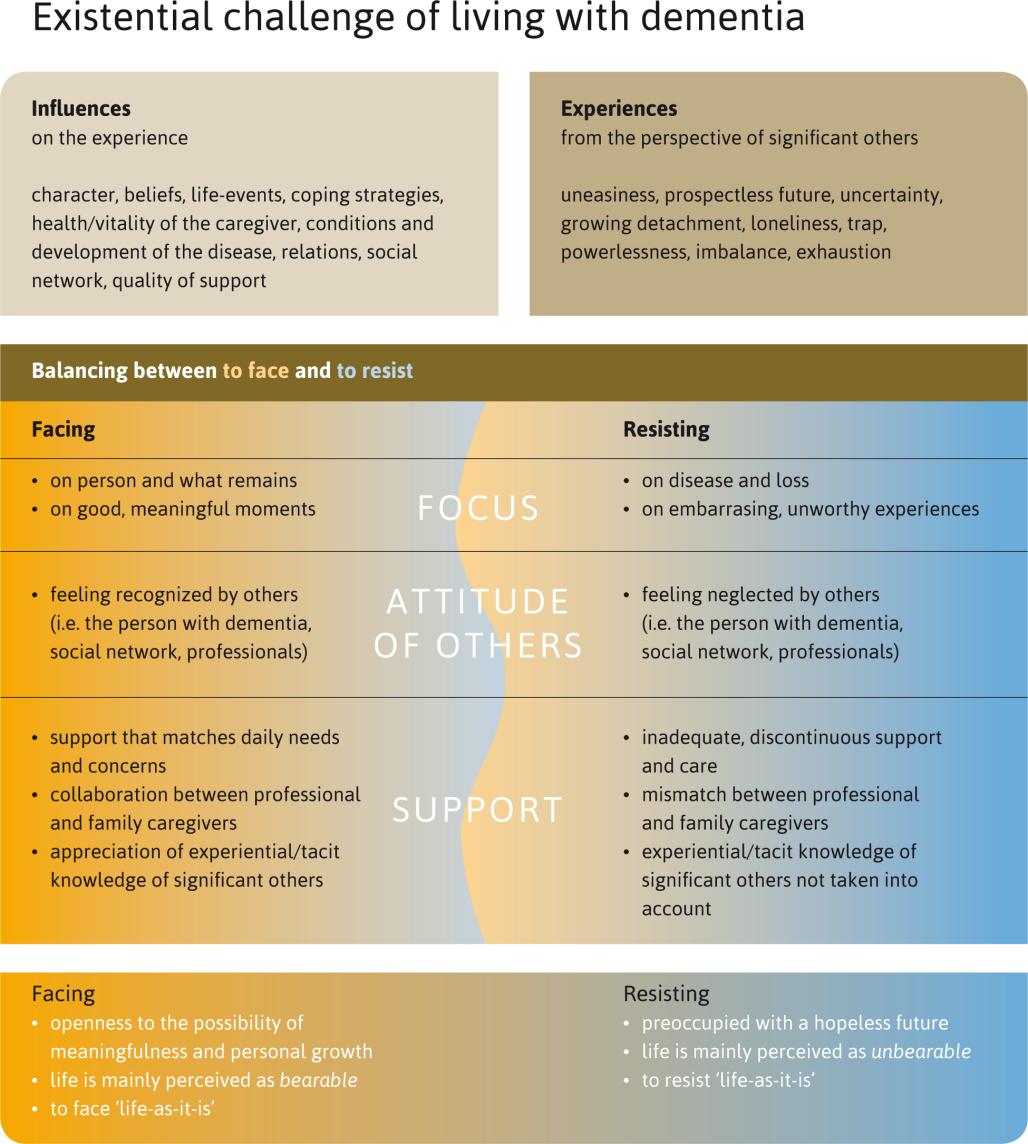
Existential challenge of living with dementia.

Based on the findings of our study, we have developed a model (Fig 1) which depicts how people handle contingency and seek a balance along the continuum of facing and resisting. The model provides an overview of the existential, balance-seeking challenges and shows various ways in which family caregivers deal with them. In this regard, one of the most interesting findings is that the art of living with dementia seems to lie in the attempt to face the ‘givens of dementia’, rather than persisting in denying, avoiding or resisting them. In other words, respondents who could face both the inevitable adversities and uncertainties in dementia, *and* the gloomy existential moods these challenges evoke (such as anxieties, grief, guilt, isolation and meaninglessness) seemed to be most capable of dealing with dementia. Confronting these experiences may lead to an existential crisis in which the meaning of life itself is at stake, but it seems that this confrontation should nevertheless not be avoided. As Boss states: “Once emotions are acknowledged and brought into the open, people are better able to minimize and manage their ambivalence.”[28] This balance-seeking process should, however, not be understood as an either-or situation (either to face or to resist). On the contrary, in many cases, participants displayed ambivalences and regularly found themselves torn between the wish to face up to the situation and the tendency to strongly resist it.

What seemed most crucial and supportive in this regard was whether the family caregiver felt recognized and appreciated by the person with dementia, other family members, professionals, and/or the wider social environment). Following from this, our study shows that resilience in the context of living with dementia should not be considered a merely individual mental ability to adapt to the circumstances, nor a set of behaviours, but should be viewed rather as a social-ecological enterprise.

### Implications for practice

Clearly, dementia profoundly affects immediate family members and close friends, especially those who are involved in the caregiving. Often they experience multiple losses and they are unable to fully mourn each loss, as the losses accumulate very quickly. They undergo considerable psychological, social and existential challenges as they witness the progression of the disease in their loved one. We argue that first and foremost, acknowledgement and recognition of this complex existential journey is what family caregivers need from professionals. Supportive interventions and good information are very important, but good care starts with deep listening and careful attention.

### Limitation of the study

While our study provides an in-depth understanding of the lifeworld of family caregivers based on rich data with a wide variety, there is at least one limitation that should be taken into account when interpreting our results. Our sample was comprised of a wide variety of family caregivers in terms of gender, age, relationship (i.e. spouses, children, and grandchildren), and different cultural backgrounds. This has led to a rich data set, which is a prerequisite for rigorous qualitative research [20, 23, 39]. However, our sample was selected through a non-random process, based on voluntary applications which means that we have only included the narratives of people who were in favour of joining our research project and sharing their stories. It is possible that individuals who volunteered to participate in this study differed from those who chose not to participate in certain ways, such as being more pro-active or experiencing less burden and grief. The study may fail to fully capture the perspectives from ‘difficult-to-reach people’ [39].

## Supporting information

S1 File Background information project. (DOCX)

S2 File Interview guide. (DOCX)
