# Supplementary material for: Entangled in uncertainty: The experience of living with dementia from the perspective of family caregivers
Source: PLoS One. 2018 Jun 13;13(6):e0198034. doi: 10.1371/journal.pone.0198034 (PMC5999274; doi:10.1371/journal.pone.0198034)
Supplement: S1 File — (DOCX) [file pone.0198034.s001.docx]

**Appendix 2 – Background information project**

The Narratives of Dementia Collection & The Experimental Laboratory Dementia

**In January 2015, we launched the *Dementieverhalenbank,* which is** a Dutch digital Narratives of Dementia Collection, in order to **preserve and share the narratives of people with dementia and their caregivers.**

**With the** Narratives of Dementia Collection**, we have several aims, namely:** to **teach** the value of deep listening**; to share supportive experiences for those living with the condition; and, by** increasing the visibility of the situation at home, to **nuance and** differentiate the dominant (sometimes disastrous) societal images of dementia **– and weave into the fabric of our society an understanding of the daily life of people living with dementia**. To this end a selection of interviews was edited into readable narratives that were anonymously published on the website (https://www.dementieweb.nl/verhalen/dementiedagboeken/) and in a book (1).

**Most importantly, the main objective of the** Narratives of Dementia Collection **is to** create a valuable archive for research purposes. By analyzing the stories, we aim to grasp the (often hidden) needs in the everyday life of people living with dementia. This provides insight into what it means to live with dementia for those diagnosed with the disease and their significant others. These insights are used to improve the care and support for people with dementia and their **significant others, and to develop new interventions (2, 3).**

Synchronous with the launch of the digital narrative collection, the *Proeftuin Sociale Benadering Dementie* was established, which is a unique experimental learning and research laboratory for the structural development, implementation and evaluation of new ideas, interventions, methods and institutional arrangements in dementia care. The main aim of this laboratory is to enable a better life for people with dementia and their close ones (3). Today, the laboratory has developed into a broad and unique collaboration between the research institute *Tao of Care*, a major healthcare institute (De KwadrantGroep), case managers, GPs, gerontologists and health insurers (4).

**References:**

1. The AM, Roolvink A, Smit M, editors. Van verhalen naar inzichten: Een selectie uit de Dementie Verhalenbank. Amsterdam: Dementie Verhalenbank; 2015.

2. van der Wedden H, Komen R, Van der Reijden I, Henning Z, The AM. Van verhalen naar inzichten: Eerste rapportage van de Dementieverhalenbank in opdracht van het Ministerie van VWS. Amsterdam: Tao of Care; 2017.

3. The AM. Dagelijks leven met dementie: een blik achter de voordeur [Daily life with dementia: looking behind the front door]. Amsterdam: Thoeris; 2017.

4. The AM, Jonkers R. Zaaien en oogsten: Proeftuin Sociale Benadering Dementie. Amsterdam: Tao of Care; 2017.
