# Supplementary material for: Entangled in uncertainty: The experience of living with dementia from the perspective of family caregivers
Source: PLoS One. 2018 Jun 13;13(6):e0198034. doi: 10.1371/journal.pone.0198034 (PMC5999274; doi:10.1371/journal.pone.0198034)
Supplement: S2 File — (DOCX) [file pone.0198034.s002.docx]

**Appendix 1 – Interview guide**

Semi-structured interviews for the *Dementieverhalenbank*-project


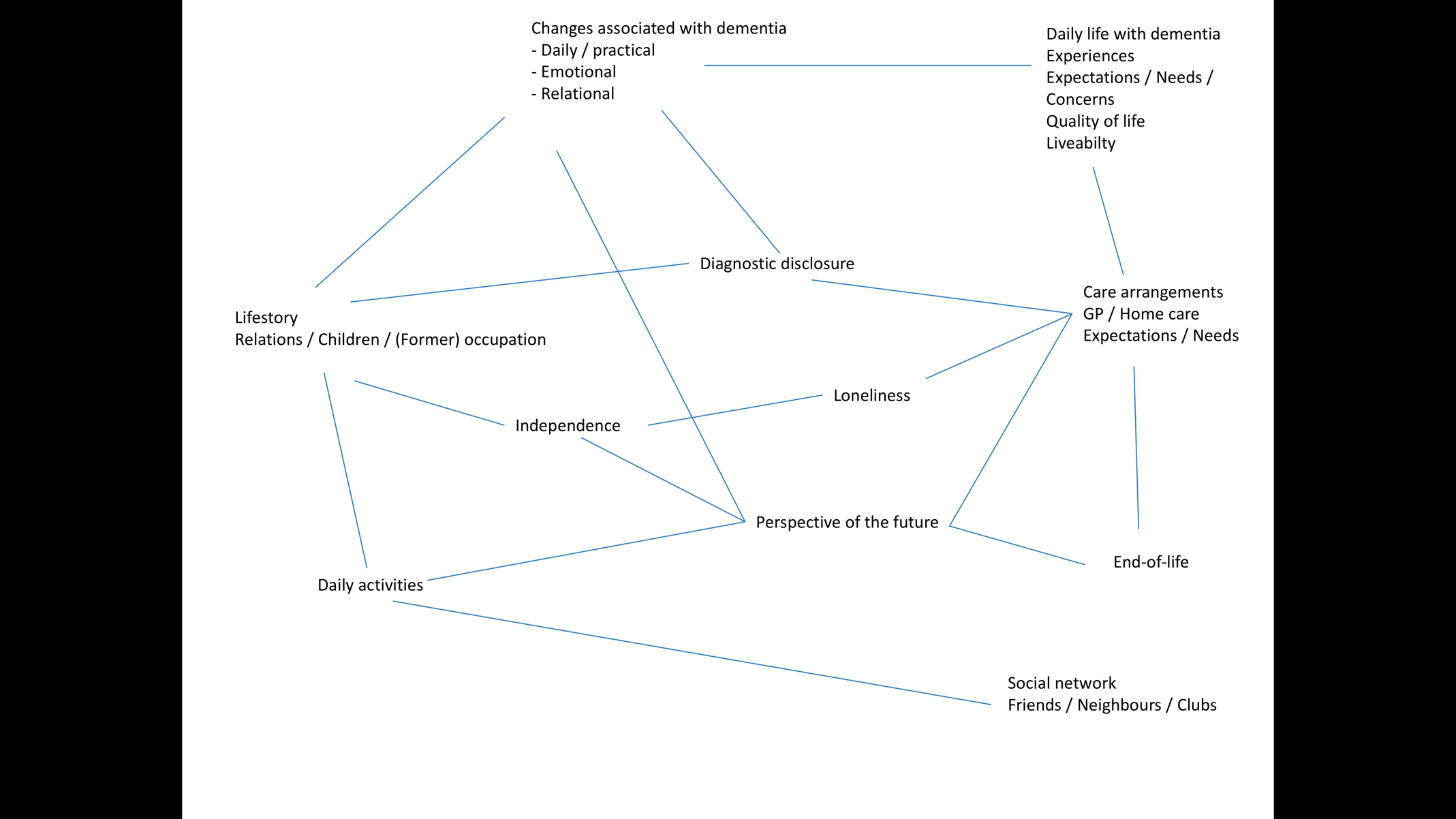


**References:**

1. Evers J, Boer Fd. Het kwalitatieve interview: kenmerken, typen en voorbereiding. In: Evers J, editor. Kwalitatief interviewen: kunst én kunde. Den Haag: Lemma; 2007.
2. Evers J, Boer Fd. Het ontwerpen van een individueel interview. In: Evers J, editor. Kwalitatief interviewen: kunst én kunde. Den Haag: Lemma; 2007.
3. Evers JC. Kwalitatief interviewen: kunst en kunde. Den Haag: LEMMA; 2007.
